# The Profiling and Identification of the Absorbed Constituents and Metabolites of Guizhi Decoction in Rat Plasma and Urine by Rapid Resolution Liquid Chromatography Combined with Quadrupole-Time-of-Flight Mass Spectrometry

**DOI:** 10.3390/ijms17091409

**Published:** 2016-09-12

**Authors:** Hongjun Xiang, Lishi Zhang, Jiannan Song, Bin Fan, Yinglan Nie, Dong Bai, Haimin Lei

**Affiliations:** 1 School of Chinese Pharmacy, Beijing University of Chinese Medicine, Beijing 100102, China; iloveygr@163.com; 2 Institute of Basic Theory for Chinese Medicine, China Academy of Chinese Medical Sciences, Beijing 100700, China; zls0423@163.com (L.Z.); sjn2003@sina.com (J.S.); 3 China Experimental Research Center, China Academy of Chinese Medical Sciences, Beijing 100700, China; binf@263.net (B.F.); nyl100@163.com (Y.N.)

**Keywords:** Guizhi decoction, rapid resolution liquid chromatography with quadrupole-time-of-flight mass spectrometry (RRLC-Q-TOF-MS), identification, metabolites

## Abstract

Guizhi decoction (GZD), a well-known traditional Chinese medicine (TCM) prescription consisting of Ramulus Cinnamomi, Radix Paeoniae Alba, Radix Glycyrrhizae, Fructus Jujubae and Rhizoma Zingiberis Recens, is usually used for the treatment of common colds, influenza, and other pyretic conditions in the clinic. However, the absorbed ingredients and metabolic compounds of GZD have not been reported. In this paper, a method incorporating rapid resolution liquid chromatography (RRLC) with quadrupole-time-of-flight mass spectrometry (Q-TOF-MS) was used to identify ingredients after oral administration of GZD. Identification of the primary components in GZD, drug-containing serum and urine samples was carried out in order to investigate the assimilation and metabolites of the decoction in vivo. By comparing the total ion chromatograms (TICs) of GZD, a total of 71 constituents were detected or characterized. By comparing TICs of blank and dosed rat plasma, a total of 15 constituents were detected and identified as prototypes according to their retention time (*t*_R_) and MS, MS/MS data. Based on this, neutral loss scans of 80 and 176 Da in samples of rat plasma and urine helped us to identify most of the metabolites. Results showed that the predominant metabolic pathways of (epi) catechin and gallic acid were sulfation, methylation, glucuronidation and dehydroxylation; the major metabolic pathways of flavone were hydrolysis, sulfation and glucuronidation. Furthermore, degradation, oxidation and ring fission were found to often occur in the metabolism process of GZD in vivo.

## 1. Introduction

Traditional Chinese medicine (TCM) prescriptions, usually made up of several medicinal herbals according to certain mass ratios guided by traditional Chinese medicine theory, have been used for centuries for the treatment of disorders or diseases in ancient and modern China [1,2,3]. It is well-accepted that a great deal of complex ingredients exist in compound Chinese traditional medicine (CCTM) and are effective through their preventive, therapeutic and synergistic effects [4,5]. Furthermore, the efficacy of a CCTM does not simply equal the sum of the efficacies of all active components [6,7]. Therefore, for the sake of investigating the material basis of the preventive and therapeutic effects of CCTM, it is crucial and necessary for us to make the absorbed constituents and metabolites clear in CCTM.

Guizhi decoction (GZD) is a common TCM formula composed of Ramulus Cinnamomi, Radix Paeoniae Alba, Radix Glycyrrhizae, Fructus Jujubae and Rhizoma Zingiberis Recens, which was recorded in a classic clinical TCM book titled Shanghan Lun (Treatise on Febrile Diseases) [8]. In the clinic, GZD is widely used in different systems of the body, including circulation, immunity, procreation, endocrine, digestion, nerves, etc. [9]. Modern pharmacological researches and the clinic experience have proved that GZD usually serve as antibacterial, analgesic, anti-anaphylaxis and antipyretic therapy as well as for the adjustment of enterokinesia, immune function, blood pressure and cardiac autonomic nervous balance [10,11,12]. However, up to now, there has been little reference to the absorbed components and metabolic compounds of GZD in the literature. Research on the Rhizoma Zingiberis Recens, an important drug of GZD, is particularly scarce.

Based on previous study [13,14,15], the constituents of GZD have been tentatively identified. Furthermore, we have focused on detecting and identifying the compounds absorbed in vivo and metabolites in GZD. Here, SD rats were chosen as the experimental animal. Drug-containing serum and urine samples were obtained. Preparation of GZD was conducted according to a certain ratio. Then, RRLC-Q-TOF-MS was used to detect the prototype compounds and metabolites after oral administration of GZD. The related metabolic investigation of GZD could provide useful information for further study.

## 2. Results

### 2.1. Analysis the Constituents of GZD

**Figure 1:**
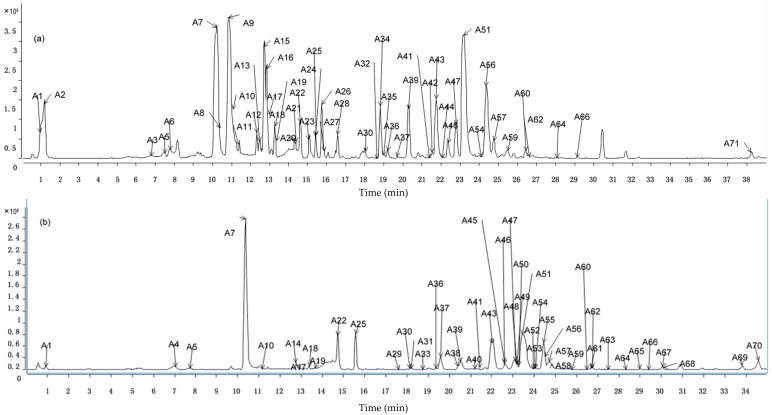
Rapid resolution liquid chromatography with quadrupole-time-of-flight mass spectrometry (RRLC-Q-TOF-MS) chromatograms of prepared Guizhi decoction (GZD). (**a**) Total ion chromatograms in negative mode; (**b**) Total ion chromatograms in positive mode.

In order to identify the constituents of Guizhi decoction (GZD), the rapid resolution liquid chromatography with quadrupole-time-of-flight mass spectrometry (RRLC-Q-TOF-MS) was used in positive and negative ion modes. Furthermore, the MS data including *t*_R_, values of *m*/*z* and MS/MS data was got from RRLC-Q-TOF-MS analysis. The total ion chromatograms (TICs) of GZD in electrospray ionization (ESI) negative and positive modes are listed in Figure 1.

The structures of most compounds in GZD were tentatively characterized by comparing their TOF-MS data, referring to related literature and comparing reference standards. Finally, a total of 71 compounds including 16 compounds originating from Radix Paeoniae Alba, one from Ramulus Cinnamomi, 33 from Radix Glycyrrhizae, four from Fructus Jujubae and 17 from Rhizoma Zingiberis Recens in GZD were detected by RRLC-Q-TOF-MS in negative or positive ion mode. In addition, 60 compounds of GZD had a known structure, and the others were isomers of part compounds from the explicit structures. The related information about identified components is listed in Table 1.

### 2.2. Analysis of Prototype Compounds in Plasma Sample

**Figure 2:**
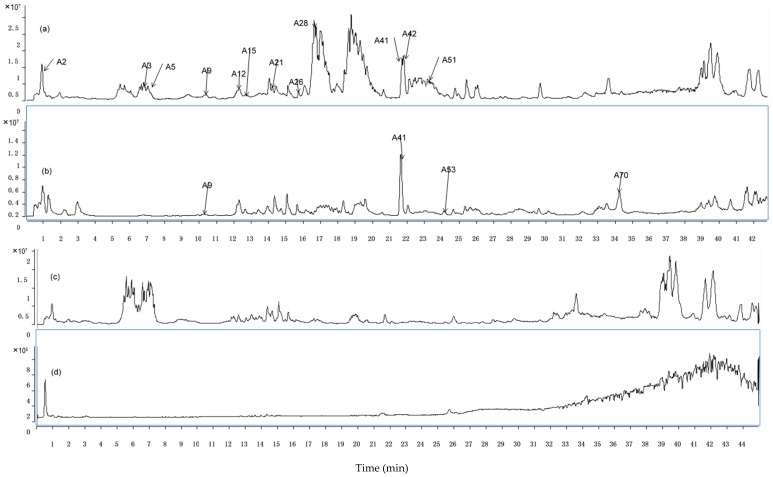
RRLC-Q-TOF-MS chromatograms of 15 prototype components in dosed and blank rat plasma. (**a**) Total ion chromatogram (TIC) of dosed rat plasma in negative mode; (**b**) TIC of dosed rat plasma in positive mode; (**c**) TIC of blank rat plasma in negative mode; and (**d**) TIC of blank rat plasma in positive mode.

For the sake of analyzing the prototype components in plasma, we compared the TICs of dosed and blank rat blood. Compared with the normal control group, a total of 15 constituents were identified from rat plasma at dosed group according to their TOF-MS data (*m*/*z*, MS/MS, t_R_ et al.). Among these 15 compounds, there are five compounds originating from Radix Paeoniae Alba (paeoniflorin, albiflorin, epicatechin, catechin and gallic acid), eight from Radix Glycyrrhizae (liquiritin, isoliquiritin, liquritigenin, isoliqurigenin, glycyrrhizic acid, naringenin-5-*O*-glucoside, naringenin-7-*O*-glucoside and formononetin), one from Ramulus Cinnamomi (cinnamic acid) and one from Rhizoma Zingiberis Recens (6-gingerol). The TICs of those 15 compounds and blank rat plasma are shown in Figure 2. In addition, prototype compounds of GZD including its related TOF-MS data can be found in Table 1.

### 2.3. Analysis of Metabolites in Blood and Urine Samples

**Figure 3:**
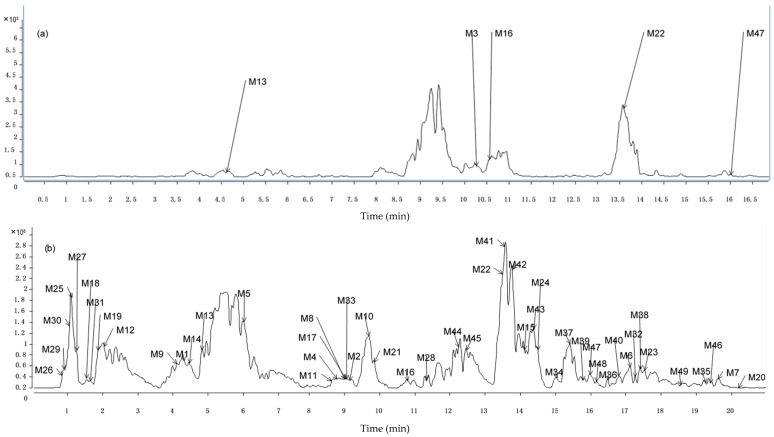
Base peak chromatograms (BPCs) with neutral loss scanning of 176 and 80 Da in negative mode to find constituents existing in (**a**) Rat plasma samples; (**b**) Rat urine samples.

As we know, after intragastric administration of the drug, the compounds originating from the drug were metabolized by intestinal bacteria in the intestine [16]. Then, they were absorbed into plasma so they can be metabolized further by all kinds of drug metabolism enzymes in liver. In principle, there are two metabolic reactions which are called phase I and phase II reactions. Through the phase I reactions including oxidation, reduction, and hydrolysis [17], the prototype components could be converted into aglycone, oxidized aglycone or reduced aglycone. After that, phase II reactions can convert the products of phase I into metabolites. In addition, the phase II reactions were focused on conjugating with glucuronide and sulfate [18,19,20,21]. In order to screen metabolites which were mainly conjugated with glucuronidation and sulfation, we use base peak chromatograms (BPCs) with neutral loss scans of 176 and 80 Da to find compounds existing in the rat plasma (Figure 3a) and urine samples (Figure 3b) in negative mode. A total of 47 peaks were identified which highly promoted the metabolite profiling of GZD. One metabolite was identified in positive mode. Comparing the TICs and, referring to references [22,23], another four metabolites were detected. The results showed that 52 components were tentatively detected as metabolites of GZD. All of the available information about the metabolites is shown in Table 2 and Table 3.

#### 2.3.1. Characterization of (epi) Catechin-Related Metabolites

**Figure 4:**
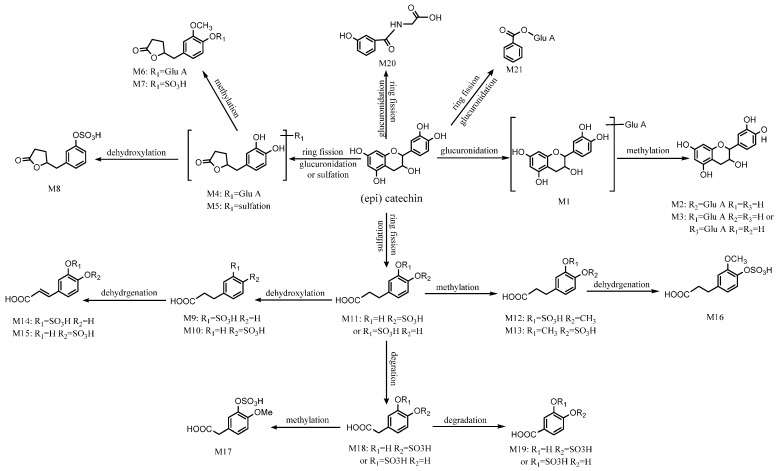
The potential metabolic profile of (epi) catechin-related metabolites.

(Epi) catechin-related metabolites are the main metabolic constituents of Radix Paeoniae Alba. Referring to Liang’s et al. researches [24] and comparing the mass spectrometry data, 21 constituents altogether were tentatively assigned in rat plasma and urine samples as metabolites, which were derived from (epi) catechin. The details are summarized in Table 2. The potential metabolism profile of (epi) catechin-related metabolites is presented in Figure 4.

#### 2.3.2. Characterization of Gallic Acid-Related Metabolites

**Figure 5:**
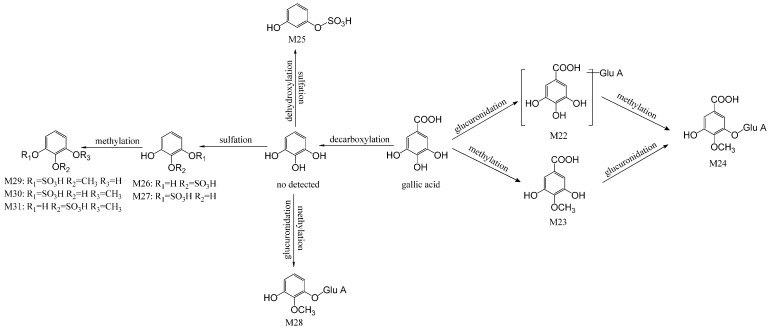
The potential metabolic profile of gallic acid-related metabolites.

The metabolites of gallic acid-related are also the main metabolites of Radix Paeoniae Alba. By referring to Liang’s et al. and Yan’s et al. studies [24,25], glucuronidation and sulfation after deglycosylation was the principal metabolic pathway of gallic acid. Ten compounds altogether were identified in animal’s plasma and urine samples as metabolites, which were derived from gallic acid. Among them, nine metabolites were characterized as glucuronide conjugates or sulfate conjugates. The available information about gallic acid-related metabolites is presented in Table 2, and a proposed metabolic pathway of gallic acid in rat is displayed in Figure 5.

#### 2.3.3. Characterization of Flavone-Related Metabolites

**Figure 6:**
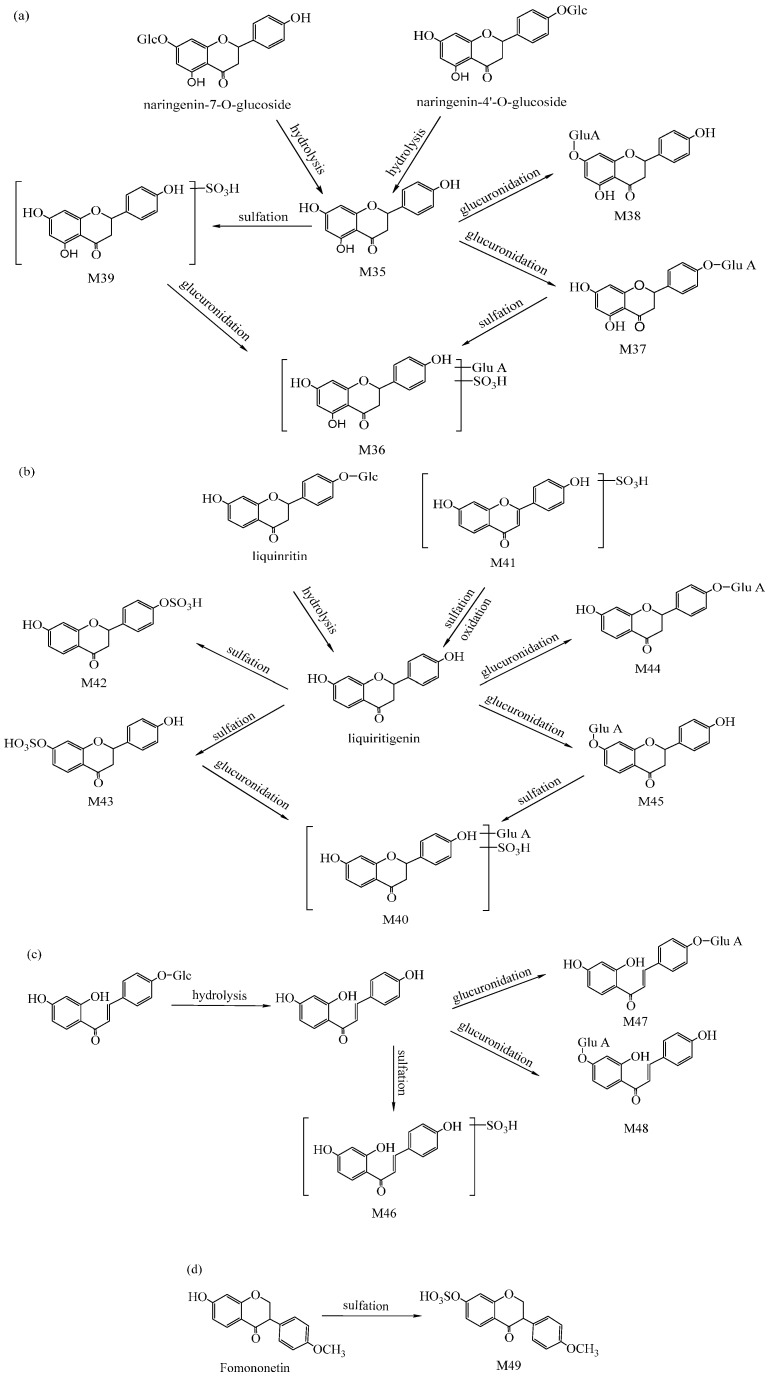
The potential metabolic profile of flavone-related metabolites. (**a**) Naringenin-*O*-glucoside-related metabolites; (**b**) Liquiritigenin-related metabolites; (**c**) Isoliquiritigenin-related metabolites; and (**d**) Formononetin-related metabolite.

The metabolites of flavone-related are the major metabolites from Radix Glycyrrhizae. There are different kinds of complicated flavone components such as naringenin-*O*-glucoside, liquiritigenin and fomononetin existing in GZD. Sixteen compounds altogether were assigned as flavone-related metabolites. Among them, six originated from naringenin-*O*-glucoside-related metabolites, six from liquiritigenin-related metabolites, three from isoliquiritigenin-related metabolites and one from fomononetin-related metabolites. The potential metabolism profile of the main flavone-related metabolites was described as shown in Figure 6, and information about all the flavone-related metabolites is listed in Table 2.

#### 2.3.4. Characterization of Other Metabolites

Paeoniflorin-related, cinnamic acid-related and gingerol-related compounds were also the most important metabolites of GZD. There are five metabolites belonging to them, among which three originated from paeoniflorin-related metabolites, one from cinnamic acid-related metabolites and one from gingerol-related metabolites. Further details can be seen in Table 2.

## 3. Discussion

In present study, we mainly discussed the identification and detection of (epi) catechin-related, gallic acid-related and flavone-related metabolites as they were found to be significant metabolites with high content in GZD. In addition, other metabolites such as gingerol-related metabolites are briefly introduced.

M1 (*t*_R_ = 4.494 min) showed [M−H]^−^ at *m*/*z* 465.0785 in a negative BPC model. The [aglycon−H]^−^ at 289.0622 was observed, conforming to a neutral loss of 176 Da (C_6_H_8_O_6_). In addition, the identification of its major fragment ion at *m*/*z* 245.0724 was in line with the related literature [26]. Thus, M1 was preliminary identified as catechin-*O*-glucuronide and its molecular formula was C_21_H_22_O_12_. M2 and M3 displayed [M−H]^−^ at *m*/*z* 479.1170 and 479.1208, respectively. Considering the appearance of the same ions at *m*/*z* 303.0860 and 175.0234, M2 and M3 are speculated to be isomers of each other. Furthermore, the loss of 176 Da indicated M2 and M3 were glucuronide conjugates. Compared to M1, catechin-*O*-glucuronide, M2 and M3 had an additional CH_2_ unit according to their high resolution mass spectrometer (HRMS) data, which determined their formula to be C_22_H_24_O_12_. Hence, M2 and M3 were identified as Methyl (epi) catechin conjugating with glucuronide. Following a previously research [27], M2 and M3 were tentatively identified as 3’-*O*-methyl (epi) catechin 5-*O*-glucuronide and 3’-*O*-methyl (epi) catechin 7- or 4’-*O*-glucuronide, respectively. In the same way, another three (epi) catechin-related metabolites conjugating with glucuronide, M4, M6 and M20 were tentatively detected and identified as 5-(3,4-dihydroxyphenyl)-γ-valerolactone glucuronide, 5-(3-methoxyl-4-hydroxyphenyl)-valerolactone glucuronide and benzoyl glucuronide, respectively. M21 (*t*_R_ = 10.089 min) was determined to be C_9_H_9_NO_4_ according to the speculation of the HRMS data, and its displayed [M−H]^−^ at *m*/*z* 194.0298. The main fragment ion at *m*/*z* 150.0562 showed a loss of 44 Da which indicated M21 might be an acid. According to a previous report [28], M21 was inferred to be 3-hydroxyhipuric acid. As for the (epi) catechin-related metabolites conjugating with sulfate, the neutral loss of molecular weight was 80 Da. According to this regulation, their HRMS data and related literature [29,30,31], we completely identified 14 compounds which were sulfate conjugates. Altogether, 21 constituents of (epi) catechin-related metabolites were identified in rat blood and urine samples.

M22 displayed [M−H]^−^ at *m*/*z* 345.1354, and its ion fragmentation was predominant at *m*/*z* 169.1232 which made clear the elimination of glucuronide residue of M22. Combining with the HRMS data, M22 was considered as gallic acid glucuronide and its molecular formula was C_13_H_14_O_11_. Likewise, the identification of another eight metabolites was carried out, including two glucuronide conjugates (M24 and M28) and six sulfate conjugates (M25, M26, M27, M30 and M31). Isomeric compounds were also examined. For the sake of distinguishing between them, a comparison of their retention times and consultation with previous literature [25,32] was performed. M23 displayed [M−H]^−^ at *m*/*z* 183.0887. Coincidently, one of the identified gallic acid-related glucuronide conjugates—M24 (4-*O*-methylgallic acid glucuronide)—showed a main ion fragment at *m*/*z* 183.0944. Moreover, the major ion fragment at *m*/*z* 183.0944 was the [aglycon−H]^−^ of M24. Therefore, M23 might be 4-*O*-methylgallic acid. The main fragment ion of M23 at *m*/*z* 139.0268 and 168.0197 confirmed that M23 was 4-*O*-methylgallic acid. In total, there were nine compounds of gallic acid-related metabolites which were tentatively detected.

M36, M37, M38, M39 were chosen as instances to elaborate the process of flavone-related identification. M36 displayed a [M−H]^−^ at *m*/*z* 527.0246. M37 and M38 showed the same [M−H]^−^ at *m*/*z* 447.1025. M39 showed a [M−H]^−^ at *m*/*z* 350.9982. M37 and M38 had the same ion fragments at *m*/*z* 271.0887 and 175.0160, which not only indicated M37 and M38 were isomeric but also confirmed that both of them conjugate with glucuronide. Based on HRMS data and related literature [25,33], M37 and M38 were tentatively identified respective as naringenin-4’-*O*-glucuronide and naringenin-7-*O*-glucuronide, and their molecular formula was C_21_H_20_O_11_. M39 had major MS/MS ions at *m*/*z* 271.0350 and 150.9829, which indicated M39 was a naringenin-related compound. The neutral loss of 80 Da showed that M39 was a sulfate conjugate. Therefore, M39 was tentatively assigned as naringenin-*O*-sulfate. M36 had its main fragment ions at *m*/*z* 447.0951, 351.0192 and 271.0628. This information suggested that M36 was conjugating with both sulfate and glucuronide. In addition, it might be a naringenin-related compound. Therefore, M36 was assigned as naringenin-*O*-glucuronide-*O*-sulfate. Altogether, there were 16 components of flavone-related metabolites which were tentatively identified.

M51 showed [M+H]^+^ at *m*/*z* 471.1015. Its major ion fragment was at *m*/*z* 295.0564 implying that M51 might be a compound conjugating glucuronide. Therefore, M51 was tentatively identified as 6-gingerol-*O*-glucuronide. Unfortunately, there are no other gingerol-related even if Rhizoma Zingiberis Recens-related metabolites were detected and identified. Considering there are many components of originated from Rhizoma Zingiberis Recens, the probable reason was that the main components of Rhizoma Zingiberis Recens were hard to metabolize. A previous report deemed that the major components of Rhizoma Zingiberis Recens are volatile oils which are not detectable and retainable with RRLC-Q-TOF-MS analysis [34]. The real reason actually remains unknown.

## 4. Materials and Methods

### 4.1. Reagents

Methanol and formic acid of HPLC grade were obtained from Merck (Darmstadt, Germany). Acetonitrile of HPLC grade was obtained from Fisher Scientific (Pittsburgh, PA, USA). Ultra-high purity water was prepared by Milli-Q system (Millipore, Billerica, MA, USA). All other chemicals were commercially available (Beijing Chemical Works, Beijing, China) and were of analytical reagent (AR) grade.

Radix Paeoniae Alba Ramulus Cinnamomi, Radix Glycyrrhizae, and Fructus Jujubae were obtained from Tong-Ren-Tang drugstore (Beijing, China). Rhizoma Zingiberis Recens was purchased from Dong-Zhi-Men vegetable market (Beijing, China). All of the crude drugs were identified by Chunsheng Liu (Academy of Traditional Chinese Medicine, Beijing University of Chinese Medicine, Beijing, China). The standards of Paeoniflorin, liquirituin, cinnamic acid, glycyrrhizic acid were got from National Institute for the Control of Pharmaceutical and Biological Products.

### 4.2. Rapid Resolution Liquid Chromatography with Quadrupole-Time-of-Flight Mass Spectrometry (RRLC-Q-TOF-MS) Analysis

RRLC-MS system is made up of an Agilent 1260 RRLC system coupled with Agilent 6520 Q-TOF mass spectrometer (6520, Aglient Technologies, Santa Clara, CA, USA). Both positive and negative ion modes were operated by an ESI source (6520, Aglient Technologies). Furthermore, full wavelength scanning analysis over an *m*/*z* range of 100–1500 was performed in positive or negative ionization mode. Data acquisition and processing were performed using Mass Hunter Qualitative Analysis B.04.00 software (Aglient Technologies).

The analytical column was operated by an Agilent ZORBAX SB-C18 (2.1 × 50 mm, 1.8 μm). The mobile phase is made up of 0.1% formic acid water (A) and acetonitrile (B). The linear elution gradient was as follows: 0–5 min, 5% B; 5–15 min, 5%–25% B; 15–25 min, 25%–40% B; 25–45 min, 40%–95% B. The injection volume was 2 mL. The flow rate was 0.3 mL/min and column temperature was at 35 °C. The conditions of the mass spectrometer under the ESI mode were as follows: ion spray voltage was 3500 V; N_2_ as drying gas and its flow rate was 10 L/min. The temperature of N_2_ 350 °C; the pressure of nebulizer was 40 spi. The collision energy was set at 20 V in initial and then changed when necessary.

### 4.3. Preparation of Guizhi Decoction (GZD)

To prepare the GZD, the crude drugs—namely, Ramulus Cinnamomi (9 g), Radix Paeoniae Alba (9 g), Radix Glycyrrhizae (6 g), Rhizoma Zingiberis Recens (9 g) and Fructus Jujubae (12 pieces)—were immersed in an eight-fold mass of distilled water for 30 min. After that, the mixture decocted for 30 min and filtered. Subsequently, a six-fold mass of distilled water was added to the mixture and decocted for another 30 min. We were combining the two filtrates and making the concentration of crude drug to 1 g/L.

### 4.4. Animal Housing Environment and Experiments

Fourteen healthy male Sprague-Dawley (SD) rats (200 ± 20 g) used in the experiments were purchased from Vital River Laboratory Animal Co., Ltd. (Beijing, China). They were maintained under standard conditions with light cycles of 12 h on and 12 h off. The room temperature and relative humidity were 23 ± 3 °C and 50% ± 10%, respectively. Principles of laboratory animal care and all protocols were in accordance with the relevant national legislation and local guidelines and were approved by Animal Care and Use Committee of the Institute of Basic Theory for Chinese Medicine, China Academy of Chinese Medical Sciences (Date: 6 August 2014; No.: 201408-16).

After 7 days’ acclimation in the metabolism cages and 12 h fasting with only water ad libitum, animals were used in experiments and randomly divided into two groups. Rats were orally administrated with GZD (10 mL/kg) once a day for two consecutive days as experimental group in Group 1. Rats were orally administrated with an equivalent volume of distilled water as normal control in Group 2. Then, 24 h urine was collected on the 9th day.

On the 9th day after the final oral administration, all rats were anaesthetized with 4% chloral hydrate, and the blood samples were collected from the portal vein. The animal serum of the same group was combined. The serum samples were obtained by centrifugation of blood at 3500 rpm for 15 min and were kept frozen at −80 °C until necessary.

### 4.5. Sample Preparation

#### 4.5.1. Preparation of GZD

To prepare the test products of GZD, GZD (prepared before, 1 g crude drug per milliliter) of 0.1 mL were placed in 10 mL volumetric flasks, diluted with methanol to volume and filtered with 0.22 µm PTFE membrane.

#### 4.5.2. Preparation of Plasma Samples

Two milliliter plasma was spiked with 6 mL methanol by vortex mixing for 30 s. Then, the mixture was immediately centrifuged for 15 min at 3500 rpm and at 4 °C to obtain the supernatant. The supernatant was shifted and evaporated to dryness. Then the residue was dissolved in 1 mL methanol. After that, the reconstituted extraction was centrifuged again for 15 min at 12,000 rpm and at 4 °C. At last, the supernatants were stored at 4 °C until RRLC-Q-TOF-MS analysis.

#### 4.5.3. Preparation of Urine Samples

Urine samples were evaporated at 65 °C to dryness. The residues were dissolved in 10 mL methanol, and the reconstituted extraction was centrifuged for 15 min at 3500 rpm and at 4 °C to obtain the supernatant. The supernatant was transferred and evaporated to dryness. Then, the residues were thoroughly dissolved in 5 mL methanol. After that, the mixture was filtered with 0.22 µm PTFE membrane. Finally, the filtrates were stored at 4 °C until RRLC-Q-TOF-MS analysis.

## 5. Conclusions

In this paper, an efficient RRLC-Q-TOF-MS method was used for separation and identification of absorbed constituents and metabolites in rat blood and urine after gavaging GZD. Altogether, 67 constituents comprising 15 prototype compounds and 52 metabolites were detected and tentatively identified in rat urine and plasma samples. In addition, 71 components altogether originating from GZD were detected or characterized. The results showed that phenolic compounds such as gingerol and shogaol were the main constituents of Rhizoma Zingiberis Recens in GZD. Cinnamic acid was the major compound of Ramulus Cinnamomi in GZD. Both phenolic compounds and cinnamic acid were found to be small polor compounds, which can be quickly absorbed into plasma. This feature indicated that GZD can be used for clinical treatment of exterior syndromes such as common colds and pyretic conditions. The major constituents of Radix Paeoniae Alba, Radix Glycyrrhizae and Fructus Jujubae were flavone and saponin. Most of them were absorbed into plasma and were metabolized by all kinds of metabolism enzymes in liver. After two-phase reaction in the liver, they were metabolized to secondary metabolites, which took effect in vivo. Analysis of metabolites showed that (epi) catechin, gallic acid and flavone were the major sources of metabolites, which originated from metabolism of GZD in vivo. Sulfation and glucuronidation were the main metabolic pathways in the metabolic process of GZD in vivo. This study systematically explored the plasma and urine metabolic profiles of GZD. The results of this study can offer essential data for deeper pharmacological and clinical studies in GZD.

## Figures and Tables

**Table 1:** Identification and detection of chemical compounds of Guizhi decoction (GZD) by rapid resolution liquid chromatography with quadrupole-time-of-flight mass spectrometry (RRLC-Q-TOF-MS).

No.	*t*_R_ (min)	Identification	Formula	Negative Ion (*m*/*z*)		Positive Ion (*m*/*z*)		Source
				Quasi-Molecular (ppm)	MS/MS (*m*/*z*)	Quasi-Molecular (ppm)	MS/MS (*m*/*z*)	
A1	0.945	cAMP	C_10_H_12_N_5_O_6_P	328.0459	134.0463	330.0590	136.0618	ZJ
A2	1.194	Gallic acid	C_7_H_6_O_5_	169.0133	125.0239	-	-	P
A3	6.804	Epicatechin	C_15_H_14_O_6_	289.0728	245.0824, 203.0730, 125.0824,109.0306	-	-	P
A4	7.035	Oxypaeoniflorin isomer I	C_23_H_28_O_12_	-	-	497.1649	197.0811, 179.0701, 151.0751, 121.0285	P
A5	7.685	Catechin	C_15_H_14_O_6_	289.0718	245.0827, 203.0720, 151.0398	291.0858	207.0669, 179.0705, 147.0432, 139.0388	P
A6	7.762	Oxypaeoniflorin	C_23_H_28_O_12_	495.1508	465.1381, 333.0985, 255.0670, 177.0554	-	-	P
A7	10.199	Albiflorin	C_23_H_28_O_11_	525.1630 [M+HCOO−H]^−^	121.0294	481.1706	197.0811	P
A8	10.391	Liquiritigenin-7-*O*-glucoside-4’-*O*-apiosyl-*O*-glucoside	C_32_H_40_O_18_	711.2131	549.1615	-	-	G
A9	10.856	Paeoniflorin	C_23_H_28_O_11_	525.1619 [M+HCOO−H]^−^	449.1453, 327.1082, 165.0541, 121.0291	-	-	P
A10	11.140	Mudanpioside I	C_23_H_28_O_11_	479.1664	121.0290	481.1703	179.0709	P
A11	11.405	Oxypaeoniflorin isomer II	C_23_H_28_O_12_	495.1513	137.0243	-	-	P
A12	12.305	Naringenin-7-*O*-glucoside	C_21_H_22_O_10_	433.1154	271.0612, 151.0034, 119.0503	-	-	G
A13	12.423	Liquiritigenin-7-*O*-glucoside-4’-*O*-apiosyl-*O*-glucoside isomer I	C_32_H_40_O_18_	711.2131	549.1615	-	-	G
A14	12.704	4-Shogoal	C_15_H_20_O_3_	-	-	249.1500	177.0920, 137.0604	ZR
A15	12.710	Liquiritin	C_21_H_22_O_9_	417.1189	255.0654	-	-	G
A16	12.810	Liquiritin-apioside	C_26_H_30_O_13_	549.1589	255.0662	-	-	G
A17	12.825	Rutin	C_27_H_30_O_16_	609.1451	301.1452	611.1645	303.0517	ZJ
A18	13.245	Galloylpaeoniflorin	C_30_H_32_O_15_	631.1660	465.1385, 313.0556, 271.0454	633.1814	315.0705, 179.0700, 153.0179, 127.0390	P
A19	13.404	Kaempferol-3-*O*-rutinoside	C_27_H_30_O_15_	593.1537	285.0394	595.1698	287.0563	ZJ
A20	14.277	Galloylpaeoniflorin isomer I	C_30_H_32_O_15_	631.1667	477.0935, 271.0604, 121.0290	-	-	P
A21	14.351	Naringenin-5-*O*-glucoside	C_21_H_22_O_10_	433.1154	271.0609, 151.0032	-	-	G
A22	14.706	Paeoniflorin isomer I	C_23_H_28_O_11_	525.1630 [M+HCOO−H]^−^	121.0291	481.1709	197.0809, 105.0339	P
A23	15.056	Lactiflorin	C_23_H_26_O_10_	507.1519 [M+HCOO−H]^−^	461.1430, 283.0631, 177.0555, 121.0292	-	-	P
A24	15.400	Lsoliquiritin-apioside	C_26_H_30_O_13_	549.1620	255.0662	-	-	G
A25	15.538	Ononin	C_22_H_22_O_9_	475.1256 [M+HCOO−H]^−^	267.0651, 252.0416	431.1336	269.0809	G
A26	15.714	Lsoliquiritin	C_21_H_22_O_9_	417.1150	225.0659, 135.0086, 119.0500	-	-	G
A27	15.813	5-hydroxylliquiritin	C_21_H_22_O_10_	433.1146	271.0607	-	-	G
A28	16.544	Liquiritigenin	C_15_H_12_O_4_	255.0671	199.0508, 135.0094	-	-	G
A29	17.605	10-Gingerdiol	C_21_H_36_O_4_	-	-	376.1810	259.1711, 137.0609	ZR
A30	18.112	Licorice saponin G2	C_42_H_62_O_17_	837.3917	351.0576	839.4062	663.3724, 469.3306	G
A31	18.125	8-Gingerdione	C_19_H_28_O_4_	-	-	321.2217	177.0923, 137.0604	ZR
A32	18.638	Pentagalloylglucose	C_41_H_32_O_26_	939.1109	-	-	-	P
A33	18.679	10-Gingerol	C_21_H_34_O_4_	-	-	373.1668 [M+Na]^+^	351.2658, 207.1017, 177.0557, 137.0605	ZR
A34	18.781	Benzoylpaeoniflorin	C_41_H_32_O_26_	629.1876, [M+HCOO−H]^−^	553.1716, 431.1349, 165.0558, 121.0291	-	-	P
A35	18.977	Apioglycyrrhizin	C_42_H_62_O_16_	821.3949	-	-	-	G
A36	19.334	Benzoylpaeoniflorin isomer	C_30_H_32_O_12_	629.1881 [M+HCOO−H]^−^	121.0293	607.1770 [M+Na]^+^	319.1183, 267.0860, 197.0808, 151.0340	P
A37	19.542	Licorice saponin A3	C_48_H_72_O_21_	983.4484	821.3958, 351.0559	985.4642	809.4310, 615.3888, 453.3359	G
A38	20.278	Acetoxyglycyrrhizin acid	C_44_H_64_O_18_	-	-	881.4165	705.3835, 511.3421	G
A39	20.503	Licorice saponin G2 isomer I	C_42_H_62_O_17_	837.3904	-	839.4058	663.3730, 469.3310	G
A40	21.153	8-Gingerdiol	C_19_H_32_O_4_	-	-	326.1860	137.0605	ZR
A41	21.354	Formononetin	C_16_H_12_O_4_	267.0661	252.0415, 223.5401	269.0806	254.0850, 225.0557	G
A42	21.510	Isoliquiritigenin	C_15_H_12_O_4_	255.0671	135.0094, 119.0498	-	-	G
A43	21.767	Licorice saponin E2	C_42_H_60_O_16_	819.3816	351.0548	-	-	G
A44	21.960	Licorice saponin G2 isomer II	C_42_H_62_O_17_	837.3907	-	839.4058	645.3617, 469.3312, 451.3203	G
A45	22.636	Licorice saponin G2 isomer III	C_42_H_62_O_17_	-	-	839.4060	487.3406, 469.3307, 451.3193	G
A46	22.636	22β-Acetoxyllicoricesaponin C2	C_44_H_64_O_17_	863.4066	-	865.4217	495.3466	G
A47	23.051	Licorice saponin G2 isomer IV	C_44_H_62_O_17_	837.3918	-	839.4069	487.3415, 469.3307, 451.3215	G
A48	23.182	6-Paradol	C_17_H_26_O_3_	-	-	279.1969	177.0940, 163.0755, 145.0661, 137.0601	ZR
A49	23.182	8-Dehydrogingerdione	C_19_H_26_O_4_	-	-	319.1886	177.0916, 163.0751, 145.0659, 137.0604	ZR
A50	23.208	6-Gingerdiol	C_17_H_28_O_4_	-	-	297.2057	177.0923, 163.0752, 137.0595	ZR
A51	23.416	Glycyrrhizic acid	C_42_H_62_O_16_	821.3952	-	823.4109	647.3780, 453.3368	G
A52	23.935	Licorice saponin G2 isomer IV	C_42_H_62_O_17_	-	-	839.4046	487.3423, 469.3307, 451.3203	G
A53	23.961	6-Gingerol	C_17_H_26_O_4_	-	-	295.1916	163.0757, 137.0605	ZR
A54	24.117	Uralenol	C_20_H_18_O_7_	369.1353	229.0864, 139.0395	371.1493	315.0874, 175.0398	G
A55	24.403	6-Shogaol	C_17_H_24_O_3_	-	-	277.1790	177.0675, 145.0647, 137.0598	ZR
A56	24.482	LS-K2	C_42_H_62_O_16_	821.3942	-	823.4411	647.3785, 453.3361	G
A57	24.664	Apioglycyrrhizin	C_42_H_62_O_16_	821.3949	-	823.4111	647.3780, 453.3362	G
A58	24.755	6-Gingerdione	C_17_H_24_O_4_	-	-	293.1773	177.0557, 145.0295, 137.0604	ZR
A59	25.756	LS-J2	C_42_H_64_O_16_	823.4130	351.0554	825.4303	453.3377	G
A60	26.458	LS-C2	C_42_H_62_O_15_	805.4300	351.0565	825.4300 [M+H2O+H]^+^	437.3435, 353.0731	G
A61	26.640	10-Gingerdione	C_21_H_32_O_4_	-	-	349.1784	177.0917, 137.0591	ZR
A62	26.718	Glycycoumarin	C_21_H_20_O_6_	367.1184	309.0402, 297.0405	369.1335	313.0725, 285.0769	G
A63	27.472	7-Shogaol	C_18_H_26_O_3_	-	-	291.1974	177.0918, 137.0606	ZR
A64	28.278	Licoricone	C_22_H_22_O_6_	381.1340	351.0869, 323.0593	383.1508	327.0872, 299.0934	G
A65	28.954	10-Dehydrogingerdione	C_21_H_30_O_4_	-	-	347.2188	177.0835, 137.0611	ZR
A66	29.344	Isoglycyrol	C_21_H_18_O_6_	365.1039	307.0247, 295.0247	367.1265	339.1254, 311.0562	G
A67	30.020	8-Shogaol	C_19_H_28_O_3_	-	-	305.2118	177.0921, 137.0604	ZR
A68	30.020	8-Gingerol	C_19_H_30_O_4_	-	-	345.2042	177.0908, 137.0596	ZR
A69	33.791	10-Shogaol	C_21_H_32_O_3_	-	-	333.2431	177.0910, 137.0595	ZR
A70	34.519	Cinnamic acid	C_9_H_8_O_2_	-	-	149.0235	121.0283	C
A71	38.121	Oleanolic acid	C_30_H_38_O_3_	455.3533	-	-	-	ZJ

Note: *t*_R_ (min): Retention time; P: Radix Paeoniae Alba; G: Radix Glycyrrhizae; C: Ramulus Cinnamomi; ZJ: Fructus Jujubae and Rhizoma; ZR: Zingiberis Recens; -: Not existed.

**Table 2:** Identification and detection of metabolites of GZD in rat plasma and urine samples by RRLC-Q-TOF-MS.

No.	*t*_R_ (min)	Identification	Formula	Urine	Plasma	Negative Ion (*m*/*z*)		Possible Original Compound	Source
						Quasi-Molecular (ppm)	MS/MS (*m*/*z*)		
M1	4.494	Catechin-*O*-glucuronide	C_21_H_22_O_12_	+	-	465.0785	289.0622, 245.0724	(Epi) catechin-related	P
M2	9.026	3’-*O*-Methyl (epi)catechin 5-*O*-glucuronide	C_21_H_24_O_12_	+	-	479.0965	303.0976, 175.0176	(Epi) catechin-related	P
M3	10.220	3’-*O*-Methyl (epi)catechin 7- or 4’-*O*-glucuronide	C_21_H_24_O_12_	-	+	479.0948	303.0876, 175.0242	(Epi) catechin-related	P
M4	8.861	5-(3,4-Dihydroxyphenyl)-γ-valerolactone glucuronide	C_17_H_20_O_10_	+	-	383.0757	207.0587, 163.0691	(Epi) catechin-related	P
M5	5.949	5-(3,4-Dihydroxyphenyl)-γ-valerolactone sulfate	C_11_H_12_O_7_S	+	-	287.0027	207.0580, 163.0685	(Epi) catechin-related	P
M6	17.003	5-(3-Methoxyl-4-hydroxyphenyl)-valerolactone glucuronide	C_18_H_22_O_10_	+	-	397.0907	221.0719	(Epi) catechin-related	P
M7	19.489	5-(3-Methoxyl-4-hydroxyphenyl)-valerolactone sulfate	C_12_H_14_O_7_S	+	-	301.0187	221.0739, 206.0503	(Epi) catechin-related	P
M8	9.305	5-(3-Hydroxyphenyl)-γ-valerolactone sulfate	C_11_H_12_O_6_S	+	-	271.0092	191.0624	(Epi) catechin-related	P
M9	4.276	4-Hydroxy phenylpropionic acid sulfate	C_9_H_10_O_6_S	+	-	244.9955	165.0596	(Epi) catechin-related	P
M10	9.549	3-Hydroxy phenylpropionic acid sulfate	C_9_H_10_O_6_S	+	-	245.0320	165.0846	(Epi) catechin-related	P
M11	8.547	3,4-Dihydroxy phenylpropionic acid sulfate	C_9_H_10_O_7_S	+	-	260.9888	181.0409, 166.0193	(Epi) catechin-related	P
M12	2.022	3-Hydroxy-4-methoxy-phenylpropionic acid sulfate	C_10_H_12_O_7_S	+	-	275.0040	195.0717	(Epi) catechin-related	P
M13	5.027	3-Methoxy-4-hydroxy-phenylpropionic acid sulfate	C_10_H_12_O_7_S	+	+	275.0042	195.0504	(Epi) catechin-related	P
M14	4.511	*m*-Coumaric acid sulfate	C_9_H_8_O_6_S	+	-	242.9793	163.0324	(Epi) catechin-related	P
M15	14.077	*p*-Coumaric acid sulfate	C_9_H_8_O_6_S	+	-	243.0173	163.0694	(Epi) catechin-related	P
M16	10.666	Ferulic acid sulfate	C_10_H_10_O_7_S	+	+	273.0259	193.0845	(Epi) catechin-related	P
M17	9.057	3-Hydroxy-4-methoxyphenylacetic acid sulfate	C_9_H_10_O_7_S	+	-	261.0254	181.0718	(Epi) catechin-related	P
M18	1.661	3,4-Dihydroxy phenylacetic acid sulfate	C_8_H_8_O_7_S	+	-	247.0111	167.0575	(Epi) catechin-related	P
M19	1.713	Protocatechuic acid-3- or -4-*O*-sulfate	C_7_H_6_O_7_S	+	-	232.9967	153.0423	(Epi) catechin-related	P
M20	20.086	Benzoyl glucuronide	C_13_H_14_O_8_	+	-	297.0939	121.0658	(Epi) catechin-related	P
M21	9.92	3-Hydroxyhipuric acid	C_9_H_9_NO_4_	+	-	194.0298	150.0462	(Epi) catechin-related	P
M22	13.393	Gallic acid glucuronide	C_13_H_14_O_11_	+	+	345.1354	169.1232	Gallic acid-related	P
M23	17.521	4-*O*-Methylgallic acid	C_8_H_8_O_5_	+	-	183.0887	168.0197, 139.0268	Gallic acid-related	P
M24	14.378	4-*O*-Methylgallic acid glucuronide	C_14_H_16_O_11_	+	-	359.1115	191.0628, 183.0944	Gallic acid-related	P
M25	1.202	2-Deoxy-pyrogallol-1-*O*-sulfate	C_6_H_6_O_5_S	+	-	188.9718	125.0010, 109.0087	Gallic acid-related	P
M26	0.845	Pyrogallol-2-*O*-sulfate	C_6_H_6_O_6_S	+	-	204.9665	125.0126	Gallic acid-related	P
M27	1.254	Pyrogallol-1-*O*-sulfate	C_6_H_6_O_6_S	+	-	204.9665	124.9982	Gallic acid-related	P
M28	30.462	2-*O*-Methylpyrogallol glucuronide	C_13_H_16_O_9_	+	-	315.2336	171.1027, 139.1119	Gallic acid-related	P
M29	0.888	2-*O*-Methylpyrogallol sulfate	C_7_H_8_O_6_S	+	-	218.9790	139.0419, 124.0168	Gallic acid-related	P
M30	1.228	1-*O*-Methylpyrogallol-3-*O*-sulfate	C_7_H_8_O_6_S	+	-	218.9809	139.0277	Gallic acid-related	P
M31	2.021	1-*O*-Methylpyrogallol-2-*O*-sulfate	C_7_H_8_O_6_S	+	-	218.9809	139.0273	Gallic acid-related	P
M32	17.146	Paeonimetabolin I glucuronide isomer I or II	C_16_H_22_O_10_	+	-	373.1311	197.1181	Paeoniflorin-related	P
M33	8.939	C_10_H_14_O_3_ sulfate	C_10_H_14_O_6_S	+	-	260.9913	181.0443	Paeoniflorin-related	P
M34	14.952	C_10_H_18_O_2_ glucuronide	C_16_H_26_O_8_	+	-	345.1354	169.1223	Paeoniflorin-related	P
M35	19.135	Naringenin	C_15_H_12_O_5_	+	-	271.0419	151.0032	Naringenin-*O*-glucoside -related	G
M36	16.194	Naringenin-*O*-glucuronide-*O*-sulfate	C_21_H_20_O_14_S	+	-	527.0264	447.0951, 351.0192, 271.0628	Naringenin-*O*-glucoside-related	G
M37	15.292	Naringenin-4’-*O*-glucuronide	C_21_H_20_O_11_	+	-	447.1025	271.0887, 175.0160	Naringenin-*O*-glucoside-related	G
M38	17.312	Naringenin-7-*O*-glucuronide	C_21_H_20_O_11_	+	-	447.1025	271.0887, 175.0160, 150.9851	Naringenin-*O*-glucoside-related	G
M39	13.133	Naringenin-*O*-sulfate	C_15_H_12_O_8_S	+	-	350.9982	271.0390, 150.9829	Naringenin-*O*-glucoside-related	G
M40	16.690	Liquiritigenin-*O*-glucuronide-*O*-sulfate	C_21_H_20_O_13_S	+	-	511.0306	431.0900, 335.0135	Liquiritigenin-related	G
M41	13.386	7,4’-Dihydroxyflavone-*O*-sulfate	C_15_H_10_O_7_S	+	-	332.9885	253.0431	Liquiritigenin-related	G
M42	13.619	Liquiritigenin-4’-*O*-sulfate	C_15_H_12_O_7_S	+	-	335.0018	255.0569, 134.9995	Liquiritigenin-related	G
M43	14.230	Liquiritigenin-7-*O*-sulfate	C_15_H_12_O_7_S	+	-	335.0018	255.0658, 135.0088	Liquiritigenin-related	G
M44	12.532	Liquiritigenin-4’-*O*-glucuronide	C_21_H_20_O_10_	+	-	431.0754	255.0498, 113.0137	Liquiritigenin-related	G
M45	12.924	Liquiritigenin-7-*O*-glucuronide	C_21_H_20_O_10_	+	-	431.0754	255.0499, 113.0132	Liquiritigenin-related	G
M46	29.631	Isoliquiritigenin-*O*-sulfate	C_15_H_12_O_7_S	+	-	335.2224	255.2111	Isoliquiritigenin-related	G
M47	16.023	Isoliquiritigenin-4’-*O*-glucuronide	C_21_H_20_O_10_	+	+	431.0754	255.0672, 113.0247	Isoliquiritigenin-related	G
M48	16.441	Isoliquiritigenin-7-*O*-glucuronide	C_21_H_20_O_10_	+	-	431.0754	255.0559, 113.0163	Isoliquiritigenin-related	G
M42	13.619	Liquiritigenin-4’-*O*-sulfate	C_15_H_12_O_7_S	+	-	335.0018	255.0569, 134.9995	Liquiritigenin-related	G
M43	14.230	Liquiritigenin-7-*O*-sulfate	C_15_H_12_O_7_S	+	-	335.0018	255.0658, 135.0088	Liquiritigenin-related	G
M44	12.532	Liquiritigenin-4’-*O*-glucuronide	C_21_H_20_O_10_	+	-	431.0754	255.0498, 113.0137	Liquiritigenin-related	G
M45	12.924	Liquiritigenin-7-*O*-glucuronide	C_21_H_20_O_10_	+	-	431.0754	255.0499, 113.0132	Liquiritigenin-related	G
M46	29.631	Isoliquiritigenin-*O*-sulfate	C_15_H_12_O_7_S	+	-	335.2224	255.2111	Isoliquiritigenin-related	G
M47	16.023	Isoliquiritigenin-4’-*O*-glucuronide	C_21_H_20_O_10_	+	+	431.0754	255.0672, 113.0247	Isoliquiritigenin-related	G
M48	16.441	Isoliquiritigenin-7-*O*-glucuronide	C_21_H_20_O_10_	+	-	431.0754	255.0559, 113.0163	Isoliquiritigenin-related	G
M49	18.463	Fomononetin-*O*-sulfate	C_16_H_12_O_7_S	+	-	347.0012	267.0637	Fomononetin-related	G

Note: *t*_R_ (min): Retention time; P: Radix Paeoniae Alba; G: Radix Glycyrrhizae; C: Ramulus Cinnamomi; ZR: Zingiberis Recens; +: Detected; -: Not detected.

**Table 3:** Table 2 supplementary.

No.	*t*_R_ (min)	Identification	Formula	Urine	Plasma	Positive Ion (*m/z*)		Possible Original Compound	Source
						Quasi-molecular (ppm)	MS/MS (*m/z*)		
M50	34.133	Glycyrrhetinic acid	C_30_H_46_O_4_	+	+	471.3544	317.2164	Glycyrrhizin-related	G
M51	23.150	6-Gingerol-*O*-glucuronide	C_23_H_34_O_10_	+	-	471.1015	295.0564	6-Gingerol-related	ZR
M52	0.524	Hippuric acid	C_9_H_8_NO_3_	+	+	178.1015	-	Cinnammic acid-related	C

Note: *t*_R_ (min): retention time; G: Radix Glycyrrhizae; C: Ramulus Cinnamomi; ZR: Zingiberis Recens; +: Detected; -: Not detected.
